# Plasmonic Metal Nanostructures Meet Triplet–Triplet Annihilation-Based Photon Upconversion Systems: Performance Improvements and Application Trends

**DOI:** 10.3390/nano13091559

**Published:** 2023-05-05

**Authors:** Jotaro Honda, Kosuke Sugawa, Hironobu Tahara, Joe Otsuki

**Affiliations:** 1 Department of Materials and Applied Chemistry, College of Science and Technology, Nihon University, Chiyoda, Tokyo 101-8308, Japan; 2 Graduate School of Engineering, Nagasaki University, Bunkyo, Nagasaki 852-8521, Japan

**Keywords:** surface plasmon resonance, photon upconversion, enhancement in annihilation, metal nanostructures

## Abstract

Improving the performance of upconversion systems based on triplet–triplet annihilation (TTA-UC) can have far-reaching implications for various fields, including solar devices, nano-bioimaging, and nanotherapy. This review focuses on the use of localized surface plasmon (LSP) resonance of metal nanostructures to enhance the performance of TTA-UC systems and explores their potential applications. After introducing the basic driving mechanism of TTA-UC and typical sensitizers used in these systems, we discuss recent studies that have utilized new sensitizers with distinct characteristics. Furthermore, we confirm that the enhancement in upconverted emission can be explained, at least in part, by the mechanism of “metal-enhanced fluorescence”, which is attributed to LSP resonance-induced fluorescence enhancement. Next, we describe selected experiments that demonstrate the enhancement in upconverted emission in plasmonic TTA-UC systems, as well as the emerging trends in their application. We present specific examples of studies in which the enhancement in upconverted emission has significantly improved the performance of photocatalysts under both sunlight and indoor lighting. Additionally, we discuss the potential for future developments in plasmonic TTA-UC systems.

## 1. Introduction

**Figure 1:**
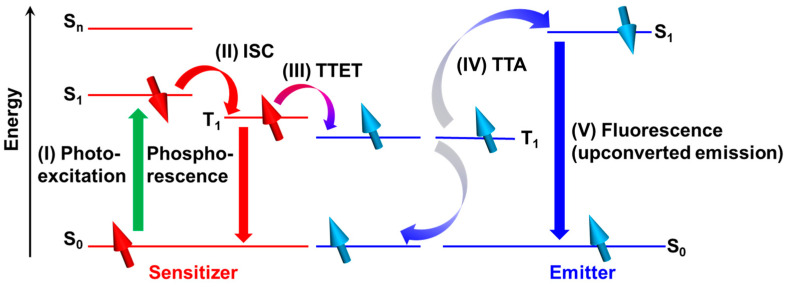
Schematic of driving mechanism of TTA-UC systems.

Upconversion techniques, including lanthanide ion-based upconversion [1,2], triplet–triplet annihilation-based upconversion (TTA-UC) [3], and two-photon absorption [4], have gained significant attention due to their ability to convert low photon-energy to high photon-energy and their wide range of potential applications. TTA-UC, first reported by Parker and Hatchard et al. [5], is particularly advantageous compared to other upconversion techniques because it can be triggered by a low-intensity and noncoherent excitation light source, such as sunlight [6,7,8,9]. Bimolecular TTA-UC systems, which typically involve a sensitizer and an emitter, can be activated by five cascade processes, as illustrated in Figure 1: (I) photoexcitation of the sensitizer molecule to the singlet-excited state, (II) generation of triplet-excited sensitizer through intersystem crossing (ISC) from the singlet-excited sensitizer, (III) generation of triplet-excited emitter through a triplet–triplet energy transfer (TTET) from the sensitizer, (IV) generation of singlet-excited emitter via a triplet–triplet annihilation (TTA) between the triplet-excited emitters, and (V) fluorescence radiation from the emitter. The choice of sensitizer–emitter combinations can vary the colors of both excitation and radiation light, ranging from the near-infrared region (NIR) to yellow, red to green, green to blue, and blue to ultraviolet (UV) [10,11]. This unique feature, coupled with the ability to be driven by sunlight, makes this optical phenomenon important for a variety of applications, including bioimaging [12,13], organic light-emitting diodes [14], and improving the performance of solar devices such as solar cells [9,15] and photocatalysts [16,17,18,19].

The TTA-UC process depends on intermolecular collision-based Dexter-type energy transfers between the sensitizer and emitter in the (III) TTET process and between the emitter and emitter in the (IV) TTA process, as shown in Figure 1. Consequently, the quantum efficiency of the upconverted emission relies on the diffusive motion of the molecules within the medium. If TTA-UC systems are intended to be used in solar devices, it is advisable to use solid-state systems for their high durability in outdoor conditions. Solid-state TTA-UC systems, where TTA-UC elements are incorporated into solid polymer hosts, are preferred for outdoor applications. However, in these systems, the quantum efficiencies are often lower than those in low-viscosity organic solvents due to the restricted mobility of the polymer hosts, which hinders the Dexter-type energy transfer for TTA-UC [20]. To improve the quantum efficiency of solid-state TTA-UC systems [21,22,23], attempts have been made to develop the TTA process via triplet exciton migration [13,16] and to use low-viscosity polymer hosts. Several reviews have been published that comprehensively cover these approaches [20,24,25]. Furthermore, the photophysical properties of the molecules in TTA-UC systems, including the optical absorption coefficient and ISC efficiency of the sensitizer and the probability of reaching the singlet-excited state after TTA and fluorescence quantum efficiency of the emitter, also significantly influence the performance of these systems. The modification of these properties can also help improve the system’s performance. Modifying the photophysical properties of molecules used in TTA-UC systems can be realized by hybridizing them with plasmonic metallic nanostructures. The localized surface plasmon (LSP) resonance of metallic nanostructures, which is caused by resonating external light with the collective oscillation of free electrons inside the metallic nanostructures, can generate highly intensified local electromagnetic fields (i.e., plasmonic electromagnetic near-fields) in the vicinity of the nanostructures [26]. Furthermore, the fluorescence of fluorescent molecules located within the electromagnetic near-fields can be significantly enhanced by two distinct mechanisms [27,28,29]: photoexcitation enhancement from the ground state and radiative process enhancement from the singlet-excited states. Typically, these enhancements decrease with an increase in the distance between the fluorescent molecules and the metallic nanostructures; hence, careful positioning of the molecules relative to the nanostructures is necessary [30]. In contrast, intentionally tuning the photophysical properties of commonly used TTA-UC elements via LSP resonance has the potential to lead to the development of innovative TTA-UC systems with enhanced performance. Furthermore, LSP resonance can be applied to various TTA-UC elements with different photoexcitation and emission wavelengths. This is because the wavelength of LSP resonance can be tuned over a wide range of wavelengths, ranging from UV to NIR, by appropriately selecting the metallic species, size, and morphology of the metallic nanostructures [31]. For example, the enhancement in photoexcitation in sensitizers via plasmonic electromagnetic near-fields, which is equivalent to the wavelength-selective enhanced light irradiation to the sensitizer, may largely contribute to the improvement in the TTA-UC performance. The relationship between upconverted emission intensity and excitation light intensity is typically quadratic, as described below [32]. Moreover, the photoexcitation enhancement in sensitizers can significantly improve the external quantum efficiency of TTA-UC systems. Using external quantum efficiency is important because while internal quantum efficiency has recently been used to evaluate TTA-UC system performance, the effective use of limited incident excitation light can only be evaluated through external quantum efficiency. Furthermore, the internal quantum efficiency may approach its theoretical limit in the near future. Additionally, the photoexcitation enhancement in sensitizers can also reduce the threshold excitation light intensity, a key figure-of-merit of TTA-UC systems, making it easier to achieve high TTA-UC efficiency with a weak light source. This review focuses on plasmonic metal nanostructure-coupled TTA-UC systems (referred to as “plasmonic TTA-UC systems”). First, this review introduces typical sensitizers utilized in TTA-UC systems. Then, the characteristic sensitizers used in recent TTA-UC systems are described. Next, we discuss how the upconverted emission enhancement in plasmonic TTA-UC systems is partially explained by the metal-enhanced fluorescence mechanism. This mechanism explains the fluorescence enhancement from individual fluorophores coupled to plasmonic metal nanostructures, providing a theoretical basis for the improved performance observed in plasmonic TTA-UC systems. Subsequently, we describe selected experiments demonstrating upconverted emission enhancement in plasmonic TTA-UC systems, as well as trends in their applications. Finally, we discuss the prospects for the future evolution of plasmonic TTA-UC systems.

## 2. Overview of Recent Studies on Sensitizers in TTA-UC Systems

**Figure 2:**
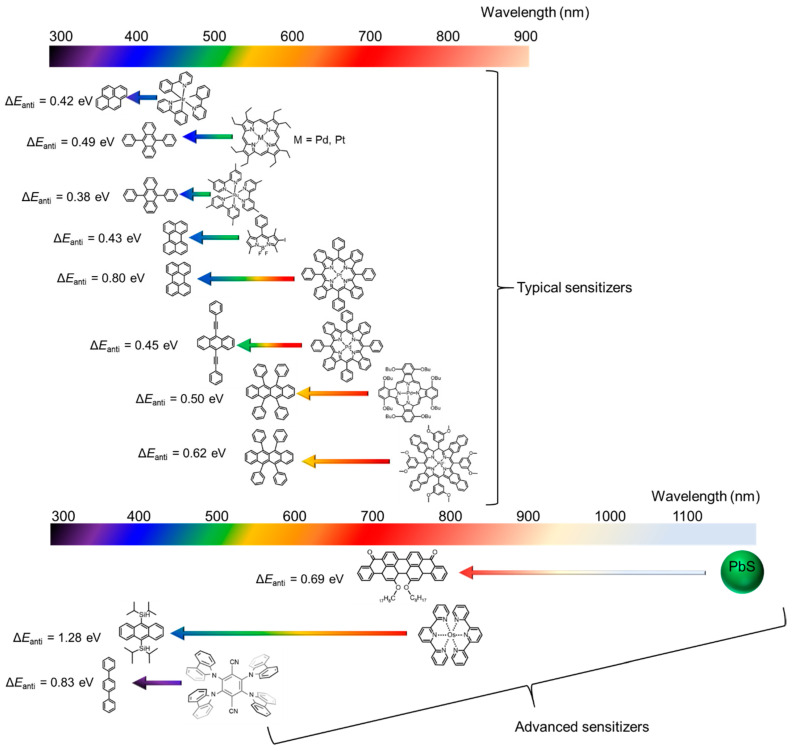
Combinations of emitter and typical sensitizer investigated so far ([11,35,36,37,38,39,40,41]), and combinations of emitter and advanced sensitizers (PbS quantum dots: [49], 4CzIPN: [43], and Os complexes: [47]).

This section presents an overview of typical sensitizer molecules utilized in TTA-UC systems and discusses current issues related to the systems. Additionally, this review covers recent studies that have explored new sensitizers in TTA-UC systems. Historically, only a limited class of emitters has been well-utilized, while recent research has expanded the use of a wide variety of photofunctional materials as sensitizers [33,34]. The upper part of Figure 2 shows the combinations of typical sensitizer and emitter molecules utilized in previously reported TTA-UC systems [11,35,36,37,38,39,40,41]. TTA-UC systems involve multiple inter- and intra-molecular optical transitions, such as ISC, TTET, and TTA, resulting in lower quantum efficiency and impaired anti-stokes shift (Δ*E*_anti_), which indicates the energy difference between the incident light and upconverted emission. Most combinations of sensitizer and emitter molecules in TTA-UC systems exhibit anti-Stokes shifts within 0.8 eV, which are smaller than those obtained for lanthanide (Ln^3+^)-doped upconversion nanoparticles (e.g., Δ*E*_anti_ = ~1.34 eV for NaYF_4_:Tm^3+^ or Em^3+^ or Ho^3+^) [42]. To address these challenges, researchers have investigated the use of thermally activated delayed fluorescence (TADF) molecules [43,44], osmium complexes [45,46,47,48], and photofunctional nanomaterials [49,50] as potential sensitizers (lower part of Figure 2).

**Figure 3:**
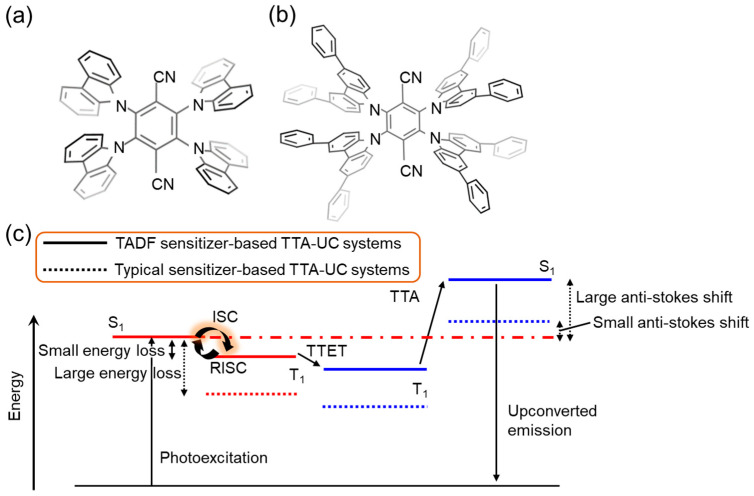
Molecular structures of (**a**) 4CzIPN, (**b**) 4CzIPN-Ph as a TADF molecule. (**c**) Energy diagram of TTA-UC systems using TADF molecule as a sensitizer (solid line) and typical sensitizer (dotted line).

Compared to common phosphorescent sensitizers, such as metalloporphyrins, which owe their high ISC efficiency to the heavy atom effect, TADF molecule sensitizers have a high ISC efficiency due to the small energy gap between their singlet- and triplet-excited states. Using TADF molecules as sensitizers in TTA-UC systems can result in a large anti-Stokes shift (Figure 3c) due to the minimized energy loss from the ISC process, in contrast to metalloporphyrins-based systems with higher ISC energy losses (e.g., platinum octaethylporphyrin (PtOEP): 0.38 eV) [51]. Recently, a TTA-UC system utilizing a typical TADF molecule, 1,2,3,5-tetrakis(carbazol-9-yl)-4,6-dicyanobenzene (4CzIPN), has been developed, which has a small ISC energy loss of 83 meV (Figure 3a,b) [52]. Nevertheless, the performance (internal quantum efficiency) of the 4CzIPN-based TTA-UC systems remains a challenge due to the competing pathways of TTET and reverse ISC from the triplet to the singlet-excited states of 4CzIPN, which are induced by the small energy gap between the two states, as shown in Figure 3c.

**Figure 4:**
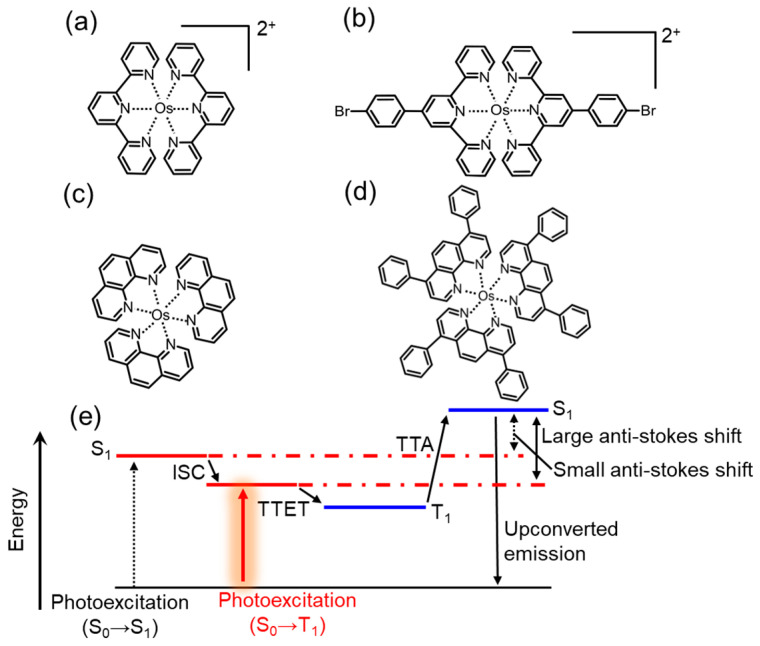
Molecular structures of Os complex sensitizer reported so far: (**a**) Os(terpyridine)_2_^2+^, (**b**) Os(bromophenyl-terpyridine)_2_^2+^, (**c**) Os(phenanthroline)_3_, and (**d**) Os(diphenyl phenanthroline)_3_. (**e**) Energy diagram of TTA-UC systems using the Os complexes.

Osmium complexes shown in Figure 4a–d possess a strong heavy atom effect that enables them to directly experience an optical transition from the ground singlet to triplet-excited states. This unique property allows for the development of TTA-UC systems that completely exclude energy losses originating from the ISC process, as shown in Figure 4e. Os(tpy)_2_^2+^ complexes, when used as a sensitizer in TTA-UC systems (Figure 4a), have demonstrated UV light emission at 425 nm under NIR excitation (724 nm), with a large anti-Stokes shift of 1.28 eV [47].

Sensitizers such as photofunctional semiconductor nanoparticles, quantum dots, and perovskite nanomaterials can be utilized in TTA-UC systems to achieve a large anti-Stokes shift, as these materials possess triplet-excited energy levels that are comparable in energy to a singlet-excited energy level [53,54]. In addition, the quantum dots have a tunable absorption peak due to the quantum size effect and exhibit high photostability. For example, TTA-UC systems driven by NIR light (>1000 nm) have been developed using PbS quantum dots, which is not possible using commonly used molecular sensitizers [49,55].

As described above, suitable sensitizers significantly improve TTA-UC system performance. However, these sensitizers commonly have an extremely low light absorption capability. Raising the concentration of sensitizers in TTA-UC systems to improve their light absorption is not a suitable approach, as this also leads to a concurrent increase in upconverted emission reabsorption by the sensitizers. This is due to the fact that the majority of sensitizers have optical absorption capabilities, even at wavelengths shorter than the excitation wavelength. The use of localized surface plasmon (LSP) resonance of metal nanoparticles can address these issues by enabling the modification of the sensitizers’ optical absorption capability at a specific wavelength.

## 3. Performance Improvement in TTA-UC Systems Utilizing Plasmonic Metal Nanostructures

### 3.1. Theoretical Prediction for Plasmon-Enhanced Upconverted Emission

**Figure 5:**
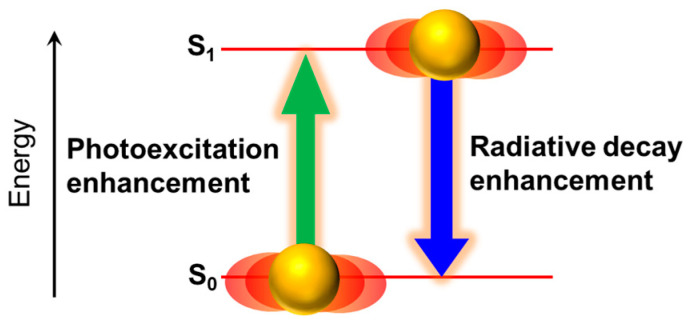
Mechanism of metal-enhanced fluorescence of fluorophores via LSP resonance.

The transition rate *γ*_i→f_ from the initial state Ψ_i_ to the final state Ψ_f_ can be expressed by Fermi’s golden rule with local enhanced filed **E** and transition electric dipole moment.

(1) $$\gamma_{i\to f}=\frac{4\pi^{2}}{h}{\left|\langle \Psi_{f}\left|\mu \cdot E\right|\Psi_{i}\rangle \right|}^{2}\rho_{f},$$

where *h*, *ρ*_f_, and **μ** are the Plank’s constant, the density of the final state, and the transition dipole moment, respectively. The mechanisms of fluorescence enhancement due to the interaction with LSP resonance in the regime of weak coupling, known as “metal-enhanced fluorescence”, are attributed to the enhancement in both photoexcitation of fluorophores and radiative decay from the singlet-excited state of fluorophores (Figure 5) because the interaction of fluorophores with plasmonic electromagnetic near-fields of metal nanostructures corresponds to the enhancement in **E** in Equation (1).

By contrast, it is currently unclear how plasmonic electromagnetic near-fields affect bimolecular TTA-UC systems that involve complicated multistep intramolecular and intermolecular optical transitions for the upconversion process. However, insights into the enhancement mechanisms of upconverted emission can be gained by considering the mechanism of metal-enhanced fluorescence. This is because TTA-UC systems typically consist of diluted sensitizer and emitter molecules in a matrix, and the interaction between these systems and LSP resonance corresponds to the weak coupling regime. This section aims to provide a theoretical explanation for the enhancement in upconverted emission through coupling with the plasmonic metal nanostructures by applying the metal-enhanced fluorescence mechanism to the radiation mechanism of TTA-UC-based upconverted emission.

The internal quantum efficiency of the upconverted emission in typical TTA-UC systems can be expressed as [56]:

(2) $$\Phi_{\mathrm{UC}}=\frac{1}{2}f\Phi_{\mathrm{ISC}}\Phi_{\mathrm{TTET}}\Phi_{\mathrm{TTA}}\Phi_{E},$$

where *f* is the probability of providing a singlet excited state after the TTA between the emitters and $\Phi_{\mathrm{ISC}}$, $\Phi_{\mathrm{TTET}}$, $\Phi_{\mathrm{TTA}}$, and $\Phi_{E}$ are the quantum efficiencies of ISC, TTET, TTA, and the emitter fluorescence, respectively. While the intrinsic photophysical properties of the sensitizer and emitter determine the value of $\Phi_{\mathrm{ISC}}$ and $\Phi_{E}$, respectively, the intermolecular energy transfers, $\Phi_{\mathrm{TTET}}$ and $\Phi_{\mathrm{TTA}}$, can be influenced by the physical properties of the surrounding matrices. For example, the energy transfer efficiencies can be influenced by the diffusive motion of the TTA-UC elements, which is strongly dependent on the viscosity of the surrounding matrix. $\Phi_{\mathrm{TTA}}$ is expressed as [56]:

(3) $$\Phi_{\mathrm{TTA}}^{0}=\frac{\gamma_{\mathrm{TT}}\left(\Phi_{\mathrm{ISC}}^{0}\Phi_{\mathrm{TTET}}^{0}\gamma_{\mathrm{ext}}^{0}I_{\mathrm{ext}}\right)}{{\left(k_{\mathrm{tot}}^{\mathrm{TE}}\right)}^{2}},$$

where the $\gamma_{\mathrm{TT}}$ is the second-order decay rate for the TTA of the triplet-excited state of the emitter and $\gamma_{\mathrm{ext}}^{0}$, $I_{\mathrm{ext}}$, and $k_{\mathrm{tot}}^{\mathrm{TE}}$ are the photoexcitation efficiency, excitation light intensity, and total decay rate from the triplet-excited emitter, respectively. The superscript “0” indicates the efficiencies obtained for the original TTA-UC systems, which are not hybridized with any plasmonic metal nanostructures. As shown in Equation (3), $\Phi_{\mathrm{TTA}}^{0}$ can be saturated to unity with a high excitation light intensity; the lowest excitation light intensity that can achieve the saturated $\Phi_{\mathrm{TTA}}^{0}$ is defined as the threshold excitation light intensity (*I*_th_). The upconverted emission intensity is expressed as:

(4) $$I_{\mathrm{UC}}^{0}=\frac{1}{2}f\Phi_{\mathrm{ISC}}^{0}\Phi_{\mathrm{TTET}}^{0}\Phi_{\mathrm{TTA}}^{0}\Phi_{E}^{0}\gamma_{\mathrm{ext}}^{0}I_{\mathrm{ext}}=\Phi_{\mathrm{UC}}^{0}\gamma_{\mathrm{ext}}^{0}I_{\mathrm{ext}}.$$

Substituting Equations (3) into (4) yields:

(5) $$I_{\mathrm{UC}}^{0}=\frac{f\gamma_{\mathrm{TT}}{\left(\Phi_{\mathrm{ISC}}^{0}\right)}^{2}{\left(\Phi_{\mathrm{TTET}}^{0}\right)}^{2}\Phi_{E}^{0}{\left(\gamma_{\mathrm{ext}}^{0}\right)}^{2}{\left(I_{\mathrm{ext}}\right)}^{2}}{2{\left(k_{\mathrm{tot}}^{\mathrm{TE}}\right)}^{2}}.$$

**Figure 6:**
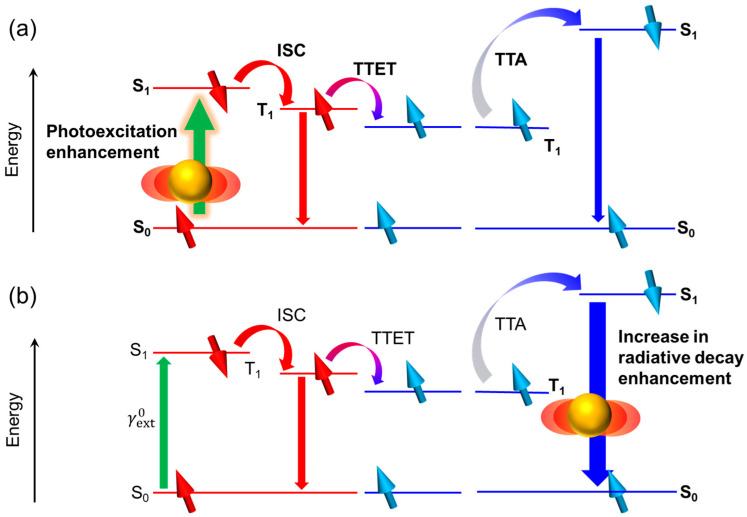
Schematic of plasmonic TTA-UC systems. Upconverted emission enhancement by (**a**) photoexcitation enhancement in sensitizer and (**b**) increase in radiative decay process of emitter. The arrows on the molecular orbitals in the figure refer to spin orientation.

As shown in Figure 6a, when the photoexcitation efficiency of a sensitizer is enhanced by the plasmonic electromagnetic near-field effect for the hybrids of the TTA-UC systems with plasmonic metal nanostructures, the quantum efficiency of TTA ($\Phi_{\mathrm{TTA}}^{\mathrm{LSPR}\left(\mathrm{ext}\right)}$) and upconverted emission intensity ($I_{\mathrm{UC}}^{\mathrm{LSP}\left(\mathrm{ext}\right)}$) are also affected by the LSP resonance and can be expressed as follows.

(6) $$\Phi_{\mathrm{TTA}}^{\mathrm{LSPR}\left(\mathrm{ext}\right)}=\frac{\gamma_{\mathrm{TT}}\left(\Phi_{\mathrm{ISC}}^{0}\Phi_{\mathrm{TTET}}^{0}\gamma_{\mathrm{ext}}^{\mathrm{LSP}\left(\mathrm{ext}\right)}I_{\mathrm{ext}}\right)}{{\left(k_{\mathrm{tot}}^{\mathrm{TE}}\right)}^{2}},$$

(7) $$I_{\mathrm{UC}}^{\mathrm{LSP}\left(\mathrm{ext}\right)}=\frac{f\gamma_{\mathrm{TT}}{\left(\Phi_{\mathrm{ISC}}^{0}\right)}^{2}{\left(\Phi_{\mathrm{TTET}}^{0}\right)}^{2}\Phi_{E}^{0}{\left(\gamma_{\mathrm{ext}}^{\mathrm{LSP}\left(\mathrm{ext}\right)}\right)}^{2}{\left(I_{\mathrm{ext}}\right)}^{2}}{2{\left(k_{\mathrm{tot}}^{\mathrm{TE}}\right)}^{2}}.$$

The parameters affected by the photoexcitation enhancement in the sensitize are indicated by the superscript “LSP(ext)”. Consequently, the enhancement factor of the upconverted emission by the photoexcitation enhancement (*EF*(ext)) is calculated as

(8) $$EF\left(\mathrm{ext}\right)=\frac{I_{\mathrm{UC}}^{\mathrm{LSP}\left(\mathrm{ext}\right)}}{I_{\mathrm{UC}}^{0}}={\left(\frac{\gamma_{\mathrm{ext}}^{\mathrm{LSP}\left(\mathrm{ext}\right)}}{\gamma_{\mathrm{ext}}^{0}}\right)}^{2}.$$

Equation (8) clearly indicates that the upconverted emission can be enhanced in proportion to the square of the photoexcitation enhancement. This enhancement effect differs from metal-enhanced fluorescence, where the fluorescence is proportionally enhanced with respect to photoexcitation enhancement. Essentially, this effect can be attributed to improvement in both internal and external quantum efficiencies. However, when $I_{\mathrm{ext}}$ exceeds *I*_th_ (i.e., when $\Phi_{\mathrm{TTA}}^{\mathrm{LSPR}\left(\mathrm{ext}\right)}$ becomes saturated), *EF*(ext) may be enhanced proportionally to the photoexcitation enhancement, similar to metal-enhanced fluorescence. In this case, the enhancement effect can be attributed to an improvement in the external quantum efficiency only. Additionally, the upconverted emission enhancement may be influenced by not only the near-field but also far-field effects associated with the LSP resonance, which can generate strong scattered light fields that extend to far distances. The extent of this contribution depends on various factors, including the morphology, size, and metallic species. Simple metal nanoparticles exhibit a scattering cross-section ($C_{\mathrm{sca}}$) that varies proportionally to $r^{6}$/$\lambda^{4}$, where *r* denotes the nanoparticle radius and *λ* represents the wavelength (Equation (9)).

(9) $$C_{\mathrm{sca}}=\frac{8\pi }{3}k^{4}r^{6}\left|\frac{\varepsilon_{m}-\varepsilon_{d}}{\varepsilon_{m}+2\varepsilon_{d}}\right|\propto \frac{r^{6}}{\lambda^{4}},$$

where $\varepsilon_{m}$ and $\varepsilon_{d}$ are the dielectric constants of the metal and surrounding medium, respectively, and *k* is the ambient wavevector. In hybrids combining TTA-UC systems and metal nanoparticles with large $C_{\mathrm{sca}}$, the photoexcitation efficiency of the sensitizer can be enhanced by extending the effective optical path length through far-field scattering [57]. Namely, in the weak coupling regime, the enhanced scattering field by plasmons from the metal acts as a secondary light source for sensitizers in the TTA-UC systems.

The upconverted emission enhancement can be induced when the radiative process enhancement from the singlet-excited fluorophores, another mechanism of metal-enhanced fluorescence, applies to the emitter in the TTA-UC elements. The radiative process enhancement can be explained by the radiating plasmon model [28]. Typically, the fluorescence quantum efficiency of the emitter ($\Phi_{E}^{0}$) without any plasmonic metal nanostructures is generally expressed as follows:

(10) $$\Phi_{E}^{0}=\frac{k_{r}}{k_{r}+k_{\mathrm{nr}}},$$

where $k_{r}$ and $k_{\mathrm{nr}}$ are the decay rates for the original radiation and nonradiation during the deactivation process from the singlet-excited emitter, respectively. The plasmonic electromagnetic near-fields can modify the quantum efficiency of the singlet-excited emitter, as shown by the following equation:

(11) $$\Phi_{E}^{\mathrm{LSP}\left(\mathrm{em}\right)}=\frac{k_{r}+k_{r}^{\mathrm{LSP}\left(\mathrm{em}\right)}}{k_{r}+k_{r}^{\mathrm{LSP}\left(\mathrm{em}\right)}+k_{\mathrm{nr}}},$$

where the superscript “LSP(em)” indicates the parameters affected by the radiative process enhancement in the singlet-excited emitter due to LSP resonance (Figure 6b). By generating plasmonic electromagnetic near-fields, a new radiative rate, $k_{r}^{\mathrm{LSP}\left(\mathrm{em}\right)}$, is added, leading to an increased overall radiative decay rate and enhanced fluorescence quantum efficiency. As a result, the modified internal quantum efficiency ($\Phi_{\mathrm{UC}}^{\mathrm{LSP}\left(\mathrm{em}\right)}$), intensity ($I_{\mathrm{UC}}^{\mathrm{LSP}\left(\mathrm{em}\right)}$), and enhancement factor ($EF\left(\mathrm{em}\right)$) of the upconverted emission can be expressed as follows:

(12) $$\Phi_{\mathrm{UC}}^{\mathrm{LSP}\left(\mathrm{em}\right)}=\frac{1}{2}f\Phi_{\mathrm{ISC}}^{0}\Phi_{\mathrm{TTET}}^{0}\Phi_{\mathrm{TTA}}^{0}\Phi_{E}^{\mathrm{LSP}\left(\mathrm{em}\right)},$$

(13) $$I_{\mathrm{UC}}^{\mathrm{LSP}\left(\mathrm{em}\right)}=\frac{1}{2}f\Phi_{\mathrm{ISC}}^{0}\Phi_{\mathrm{TTET}}^{0}\Phi_{\mathrm{TTA}}^{0}\Phi_{E}^{\mathrm{LSP}\left(\mathrm{em}\right)}\gamma_{\mathrm{ext}}^{0}I_{\mathrm{ext}},$$

(14) $$EF\left(\mathrm{em}\right)=\frac{I_{\mathrm{UC}}^{\mathrm{LSP}\left(\mathrm{em}\right)}}{I_{\mathrm{UC}}^{0}}=\frac{\Phi_{E}^{\mathrm{LSP}}}{\Phi_{E}^{0}}.$$

Unlike the photoexcitation enhancement in the sensitizer, the enhancement in upconverted emission due to radiative process improvement can be attributed solely to an increase in internal quantum efficiency, as indicated by these equations. Since TTA-UC systems typically use fluorescent molecules with high quantum efficiencies, the enhancement in the upconverted emission due to an improved radiative process may be insignificant. However, in TTA-UC systems incorporated into solid polymer hosts, there is an intrinsic trade-off between $\Phi_{\mathrm{TTA}}^{0}$ and the fluorescence quantum efficiency. High-concentration emitter systems can improve Dexter-type energy transfer through frequent intermolecular collisions, even in solid polymer hosts with limited diffusive motion. However, concentration quenching induced in the high-concentration emitter systems attenuates the fluorescence quantum efficiency. Therefore, plasmonic electromagnetic near-fields that improve the radiative process can be useful enhancement agents, particularly in solid polymer hosts.

On the other hand, metal nanostructures are also known to induce fluorescence quenching through Dexter-like (i.e., electron transfer quenching) and/or Förster-like energy transfers, which can conflict with fluorescence enhancement. The dominance of the quenching effect in plasmonic TTA-UC systems, affecting both the sensitizer and/or emitter, can markedly decrease the quantum efficiencies of ISC, TTET, TTA, and the fluorescence of the emitter.

### 3.2. Plasmonic TTA-UC Systems

Interaction between bimolecular TTA-UC systems, characterized by varying morphologies, scales, and physical properties, and metal nanostructures possessing various morphologies and metallic species, can induce the enhancement in upconverted emission through plasmonic electromagnetic near-field effects. Therefore, to achieve efficient emission enhancement and suppress conflicting quenching due to metal nanostructures, numerous plasmonic TTA-UC systems have been developed. This section describes some experiments that demonstrate this phenomenon.

**Figure 7:**
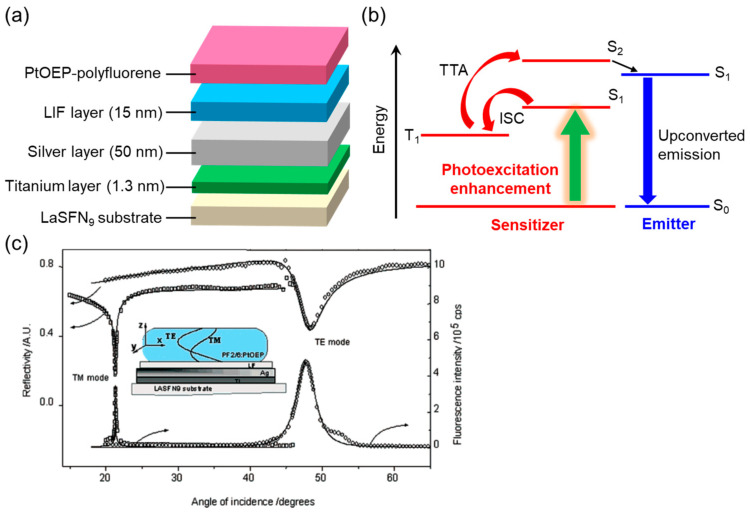
SPP-enhanced TTA-UC systems: (**a**) schematic of SPP-enhanced upconverted emission systems, (**b**) suggested mechanism of TTA-UC, and (**c**) incident angle dependence of reflectivity and upconverted emission intensity (reprinted with permission from ref. [58]. Copyright 2005 American Chemical Society).

Resonance enhancement in upconverted emission was previously reported through the use of propagating surface plasmon resonance (surface plasmon polariton: SPP) excited on a silver planar substrate based on the Kretschmann configuration, predating LSP-based TTA-UC systems [58]. To excite the SPP, a LaSFN_9_ substrate was first cleaned and then coated with a titanium layer (thickness: 1.3 nm) as an adhesive layer, a silver layer (thickness: 50 nm), and a LiF layer as a quenching-suppressing layer (Figure 7a). The substrate was then coupled with an optical prism based on the Kretschmann configuration. Spin-coating was used to prepare TTA-UC thin solid films on the substrate, which consisted of polyfluorene as a blue-emitting conjugated polymer blended with PtOEP as a sensitizer. The radiation mechanism (Figure 7b) for the upconverted emission, as claimed by the authors, differs from the typical mechanism shown in Figure 1. In this case, the upconverted emission was generated through energy transfer from a higher-order singlet (S_2_ level)-excited PtOEP, which was produced by TTA between triplet-excited PtOEPs, to the polyfluorene. Under the resonance condition induced by excitation with s-polarized light, the upconverted emission was significantly enhanced (>100 times) compared to that under the nonresonant conditions. The effective photoexcitation enhancement in PtOEP was attributed to the enhanced electromagnetic fields of the evanescent waves of SPP (Figure 7a), which resulted in the observed enhancement in upconverted emission.

**Figure 8:**
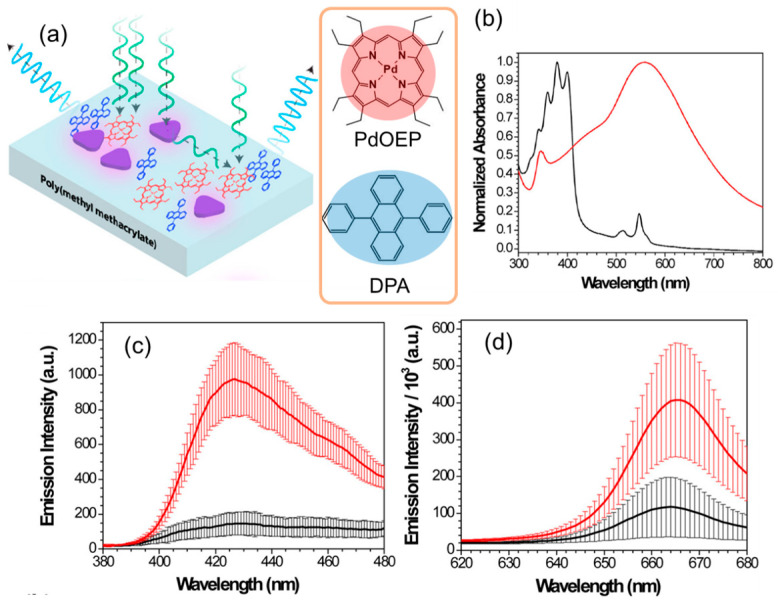
Plasmonic TTA-UC systems consisting of PdOEP, DPA, and silver nanoplates: (**a**) schematic of plasmonic TTA-UC systems, (**b**) normalized absorption spectra of DPA/PdOEP (black line) and silver nanoplates (red line) in PMMA films, (**c**) upconverted emission spectra from the systems in the presence (red line) and absence (black line) of silver nanoplates, (**d**) phosphorescence spectra from the systems in the presence (red line) and absence (black line) of silver nanoplates (reprinted with permission from ref. [59]. Copyright 2014 American Chemical Society).

In an experimental demonstration by Poorkazem et al. [59], the first evidence of enhancement in upconverted emission by LSP resonance was reported. Poly(methyl methacrylate) (PMMA) matrix-based TTA-UC films, comprising silver nanoplates with a diameter of 13 ± 2 nm, palladium octaethylporphyrin (PdOEP) as a sensitizer, and 9,10-diphenylanthracene (DPA) as an emitter, were prepared on a glass substrate by spin-coating. The schematic of these systems is shown in Figure 8a. The silver nanoplates formed stable aggregates in the solid matrix, and the LSP resonance wavelength coincided with the Q-band of PdOEP, which corresponds to the upconversion excitation wavelength (Figure 8b). As a result, both the upconverted emission (Figure 8c) and phosphorescence (Figure 8d) from the systems were enhanced 6.6 and 3.5 times, respectively, due to the enhanced photoexcitation of PdOEP by the enhanced electromagnetic near-fields of the silver nanoplates. The difference in the enhancement factors can be attributed to the nonlinear response to the number of absorbed photons for upconverted emission and the linear response for phosphorescence. These results correlate qualitatively with Equation (8), described in Section 3.1. Furthermore, the upconverted emission was enhanced up to 8.5 times by adjusting the density of the silver nanoplates encapsulated in PMMA.

**Figure 9:**
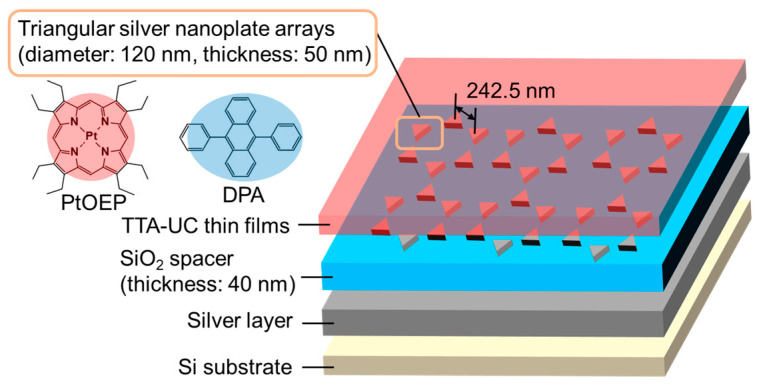
Schematic of MIM gap plasmon resonators-based TTA-UC systems.

Stronger plasmonic electromagnetic near-fields, which act on the photoexcitation process of the sensitizer, can lead to greater enhancement in upconversion [60]. The plasmonic metal nanostructure morphology plays a crucial role in dictating the strength of the electromagnetic fields. The metal–insulator–metal structures, known as MIM gap plasmon resonators, can result in constructive interference that causes standing-wave resonances. These resonances produce electromagnetic fields stronger than those of simple LSP and SPP resonances. Therefore, to achieve stronger enhancement in upconverted emission, MIM structures were employed. These structures consisted of arrays of triangular silver nanoplates (diameter: 120 nm; thickness: 50 nm), SiO_2_ spacer, and silver planar substrate. The schematic of these systems is shown in Figure 9. The plasmon absorption was observed to be the strongest when the thickness of the SiO_2_ spacer was set to 40 nm. In addition, the resonance wavelength had a significant overlap with the photoexcitation wavelength of the sensitizer (PtOEP). The 3D electromagnetic field simulation predicted the generation of strong electromagnetic fields around the triangular silver nanoplates. As a result, the TTA-UC thin films that included PtOEP and an emitter (DPA) positioned atop the MIM structures exhibited significant enhancement in upconverted emission (approximately 75 times). The upconversion enhancement was attributed to an efficient photoexcitation enhancement in PtOEP on the gap plasmon resonators. This, in turn, led to an enhancement in the TTA process between the triplet-excited DPAs, leading to more efficient upconverted emission.

**Figure 10:**
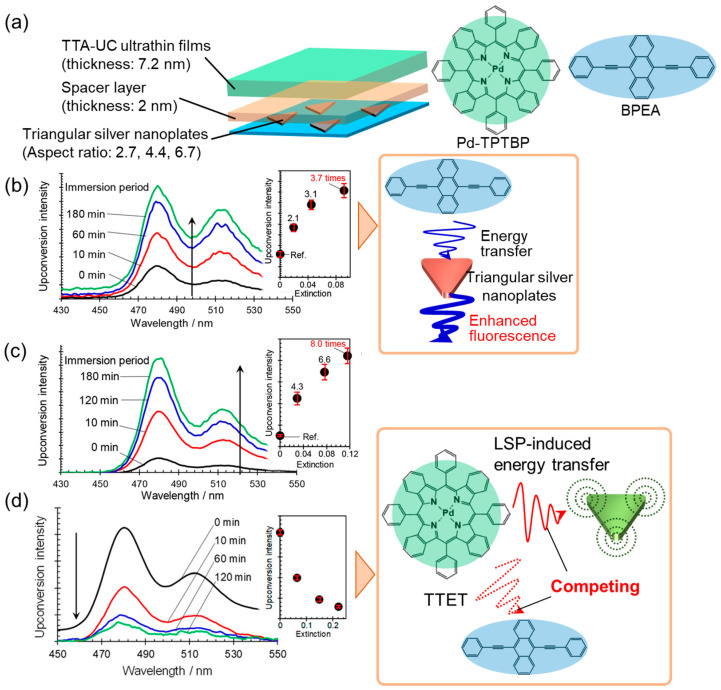
Wavelength dependence of the LSP resonance on the upconverted emission behavior. (**a**) Schematic of hybrids of triangular silver nanoplates and TTA-UC ultrathin films. Upconverted emission from the plasmonic TTA-UC systems with triangular silver nanoplates with aspect ratios of (**b**) 2.7, (**c**) 4.4, and (**d**) 6.7 (reprinted with permission from ref. [61]. Copyright 2018 American Chemical Society).

An investigation into the effect of the wavelength dependence of the LSP resonance on upconverted emission behavior was recently conducted, utilizing hierarchical hybrids of plasmonic metal nanoparticles and TTA-UC ultrathin films [61]. The ultrathin films (thickness: 7.2 ± 0.6 nm), composed of palladium (II) tetraphenyltetrabenzoporphyrin (Pd-TPTBP) as a sensitizer, 9,10-bis-(phenylethynyl)anthracene (BPEA) as an emitter, and ethylene oxide–epichlorohydrin copolymer as a host matrix were coated onto glass substrates pre-modified with triangular silver nanoplates (Figure 10a). The LSP resonance wavelength can be tuned by adjusting the aspect ratio (edge length/thickness) over a wide range of wavelengths, spanning from the visible to NIR regions. The hierarchical hybrids prepared using triangular silver nanoplates with an aspect ratio of 2.7, of which the main LSP resonance band was tuned to the BPEA fluorescence wavelengths (470–530 nm), exhibited a significant enhancement in upconverted emission (3.7 times) at the highest coverage of triangular silver nanoplates (Figure 10b). The fluorescence quantum efficiency of BPEA in the host matrix was 0.07, significantly lower than its typical efficiency range of 0.8–1 in a solution. Consequently, the observed enhancement in upconverted emission was attributed to an improvement in the fluorescence quantum efficiency of BPEA, which was induced by the energy transfer from the singlet-excited BPEA to the LSP. Hybrids prepared with triangular silver nanoplates having an aspect ratio of 4.4 exhibited a remarkable 8.0-fold enhancement in upconverted emission (Figure 10c). The primary mechanism for enhancing upconverted emission was attributed to the photoexcitation enhancement in Pd-TPTBP because the LSP resonance overlapped spectrally with the excitation wavelength, as discussed earlier. It is important to note that the *I*_th_ of the TTA-UC ultrathin films decreased significantly by 93%, from 3500 to 240 mW/cm^2^, upon hybridization with the triangular silver nanoplates. Equation (15) shows the *I*_th_ of the normal (i.e., nonplasmonic) TTA-UC systems [56], which can be modified by photoexcitation enhancement, as follows:

(15) $$I_{\mathrm{th}}^{0}=\frac{2{\left(k_{\mathrm{tot}}^{\mathrm{TE}}\right)}^{2}}{\gamma_{\mathrm{TT}}\gamma_{\mathrm{ext}}^{0}\Phi_{\mathrm{TTET}}},$$

(16) $$I_{\mathrm{th}}^{\mathrm{LSP}}=\frac{2{\left(k_{\mathrm{tot}}^{\mathrm{TE}}\right)}^{2}}{\gamma_{\mathrm{TT}}\gamma_{\mathrm{ext}}^{\mathrm{LSP}\left(\mathrm{ext}\right)}\Phi_{\mathrm{TTET}}},$$

where $I_{\mathrm{th}}^{0}$, $I_{\mathrm{th}}^{\mathrm{LSP}}$, and $k_{\mathrm{tot}}^{\mathrm{TE}}$ represent the threshold excitation light intensity from the nonplasmonic and plasmonic TTA-UC systems, and the total decay rate from the triplet-excited emitter, respectively. Experimental evidence demonstrated that the photoexcitation enhancement ($\gamma_{\mathrm{ext}}^{\mathrm{LSP}\left(\mathrm{ext}\right)}$ > $\gamma_{\mathrm{ext}}^{0}$) led to a decrease in the *I*_th_. Conversely, the triangular silver nanoplates with an aspect ratio of 6.7, whose LSP resonance spectrally overlapped with the Pd-TPTBP phosphorescence wavelengths, resulted in a notable attenuation rather than enhancement in the upconverted emission (Figure 10d). Furthermore, the observed attenuation in the upconverted emission was found to be associated with an enhancement in the Pd-TPTBP phosphorescence and a corresponding reduction in its lifetime. These results suggest that the dominant energy transferred from the triplet-excited Pd-TPTBP to the LSP rather than to the emitter, resulting in an attenuated upconverted emission. These results should provide important design guidelines for the development of optimal plasmonic TTA-UC systems.

**Figure 11:**
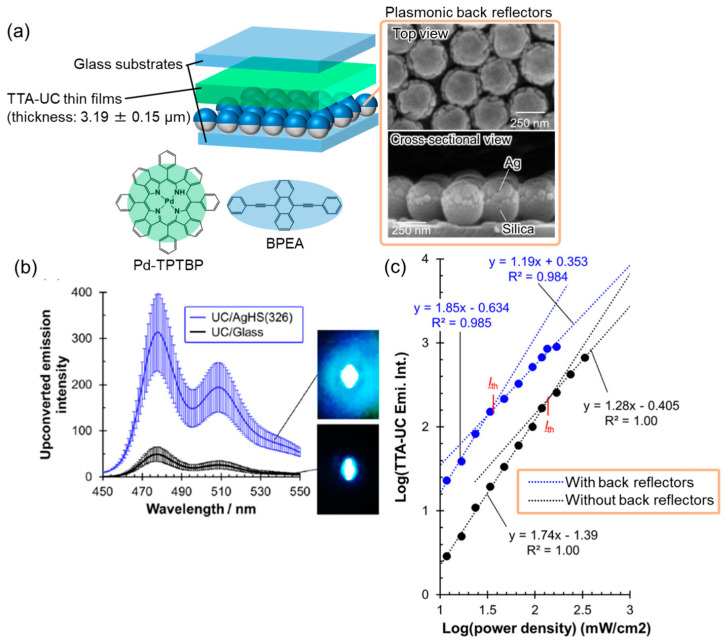
Plasmonic back-reflectors-based TTA-UC systems. (**a**) Schematic of plasmonic TTA-UC systems and SEM images of plasmonic back reflectors. (**b**) Upconverted emission spectra with and without plasmonic back reflectors. (**c**) Double logarithm plot of excitation laser intensity and upconverted emission for samples with and without plasmonic back reflectors (reprinted with permission from ref. [63]. Copyright 2021 American Chemical Society).

The plasmonic electromagnetic near-fields only affect the TTA-UC elements located in the nanospace surrounding the metal nanostructures. In contrast, the far-field scattering induced by LSP can affect the TTA-UC elements located at a distance from the metal nanostructures [62]. Therefore, the use of plasmonic back reflectors to achieve strong backward scattering for improving the performance of TTA-UC solid systems was investigated [63]. To prepare the plasmonic back reflectors, thin layers of Ag (50 nm in thickness) were deposited onto the surface of 2D colloidal crystals consisting of silica particles with a diameter of 326 nm (Figure 11a). The TTA-UC films consisting of a polyurethane rubber (Clear Flex^®^) host incorporated with Pd-TPTBP and BPEA were modified onto the plasmonic back reflector surface. The TTA-UC thin films were 3.19 ± 0.15 μm thick, which is significantly larger than the spatial extent of the plasmonic electromagnetic near-fields at the nanoscale. The reflectance spectrum of the hybrids exhibited an LSP resonance dip at the wavelength corresponding to the Q-band of Pd-TPTBP. The upconverted emission was enhanced 6.3 times, compared with that of the TTA-UC films without the back reflectors; the enhancement was confirmed by the naked eye (Figure 11b). Additionally, the presence of the back reflectors resulted in a 77% decrease in *I*_th_, as shown in Figure 11c. The enhancement factor remained consistent even after introducing optically inert interlayers with a thickness of 2–8 nm between the back reflectors and TTA-UC films, suggesting that the emission enhancement was primarily due to far-field scattering rather than near-field effects. The improved performance of the Pd-TPTBP-based TTA-UC films was attributed to the enhanced photoexcitation of the sensitizer, which was achieved by elongating the excitation light path within the films through the strong backward scattering of incident light induced by LSP. Moreover, even the low-cost aluminum plasmonic back reflectors were found to enhance the upconverted emission by 3.5 times.

**Figure 12:**
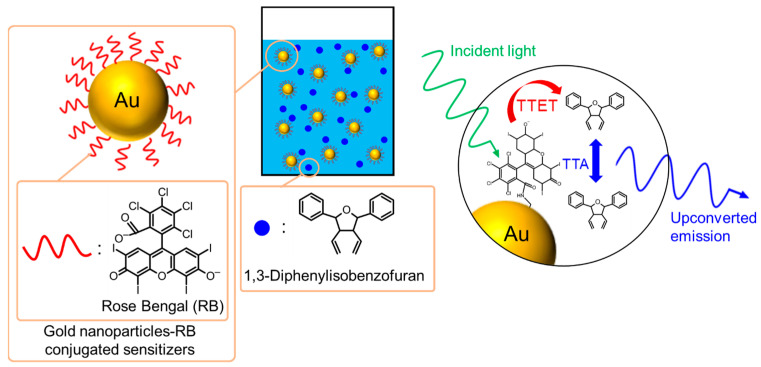
Gold nanoparticles-based plasmonic TTA-UC systems in solution.

The above-mentioned improvements in the upconverted emission were achieved through the hybridization of solid-state TTA-UC systems with plasmonic metal nanostructures. By contrast, liquid-state plasmonic TTA-UC systems have also been developed by dispersing hybrids of metal nanoparticles and sensitizers or emitters in solution. In such systems, the estimation of the enhancement mechanism is more reliable since the molecular species in close proximity to the metallic nanoparticles are known in advance. A plasmonic TTA-UC system in a liquid state was reported, in which gold nanoparticles-Rose Bengal (RB) conjugated sensitizers were dispersed in *N*,*N*-dimethylformamide with 1,3-diphenylisobenzofuran serving as the emitter [64]. A schematic of the plasmonic TTA-UC system in the liquid state is shown in Figure 12. The upconverted emission resulting from TTA between triplet-excited emitters was enhanced 1.6 times, attributed to the presence of gold nanoparticles. The authors suggest that the enhancement mechanisms may be attributed not only to the photoexcitation enhancement but also to the increased ISC efficiency of RB. This increase in ISC efficiency could be due to the promotion of the mixing of electronic opposite parities by inhomogeneous electromagnetic fields of gold nanoparticles. Interestingly, the fluorescence of RB was reduced along with the enhancement in the upconverted emission. In addition, the longer upconverted emission lifetime from the plasmonic TTA-UC systems may be a factor that promotes TTET efficiency from the triplet-excited RB to the emitter. Additionally, the authors reported a 13-fold increase in the singlet oxygen efficiency generated through the heterogeneous TTA between the triplet-excited RB and oxygen in the ground triplet state, which was attributed to the LSP resonance in the gold nanoparticles–RB conjugates. The enhancement was attributed to the efficient enhancement in photoexcitation, ISC, and TTET. Moreover, the plasmonic TTA-UC systems in the liquid state have also been prepared using conjugates composed of polymeric emitters modified with silver nanoparticles [65].

### 3.3. Application Trends in Plasmonic TTA-UC Systems

In contrast to other upconversion systems, the TTA-UC systems have the potential to be powered by low-intensity, noncoherent sources of light such as indoor lighting or sunlight. This unique feature makes them promising technology for enhancing the performance of solar devices and photocatalysts used indoors. The enhancement in upconverted emission through LSP is expected to significantly improve the performance. This section presents the performance enhancement through the use of plasmonic TTA-UC systems.

**Figure 13:**
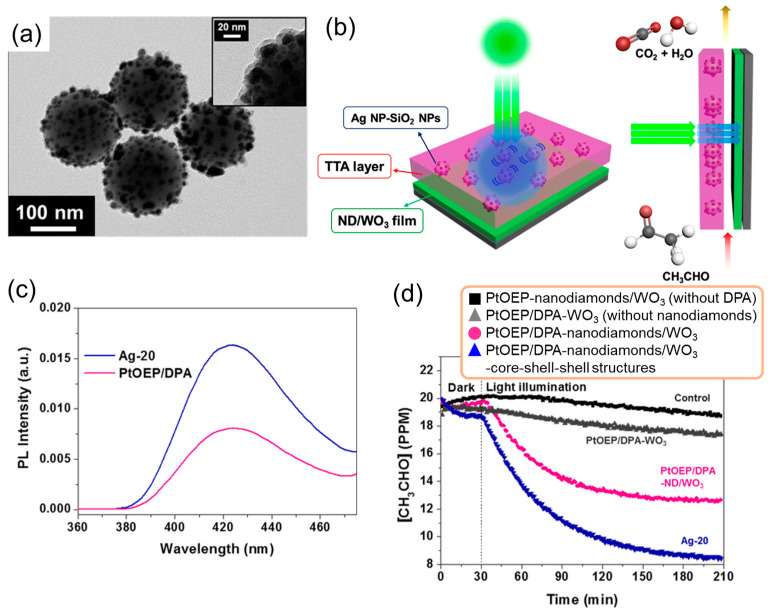
Plasmonic TTA-UC-based photocatalytic systems for decomposition of acetaldehyde. (**a**) TEM image of core(SiO_2_)–shell(silver nanoparticle assemblies)–shell(silica thin films) structures. (**b**) Schematic of plasmonic TTA-UC-based photocatalytic systems. (**c**) Upconverted emission spectra from the TTA-UC thin films in the presence (blue line) and absence (pink line) of the core–shell–shell structures. (**d**) Time profiles of the photocatalytic degradation of acetaldehyde over the course of a 3 h period with hybridized systems of TTA-UC thin films and photocatalytically active thin films (reprinted with permission from ref. [66]. Copyright 2016 American Chemical Society).

Kim et al. utilized a plasmonic TTA-UC system to photocatalytically decompose indoor air pollutants such as acetaldehyde [66]. The plasmonic TTA-UC thin films were prepared by incorporating a PtOEP sensitizer, DPA emitter, and metal nanostructures in rubbery polyurethane, which enabled efficient intermolecular energy transfers. Core–shell–shell metal nanostructures were used, which consisted of SiO_2_ particles as cores (diameter, 150–200 nm), silver nanoparticle assemblies as inner shells, and silica thin films as outer shells (thickness, 5 nm), as shown in Figure 13a. The TTA-UC-based photocatalytic systems were prepared by integrating photocatalytically active thin films, specifically WO_3_ loaded with cocatalyst nanodiamonds, with two plasmonic TTA-UC thin films, as shown in Figure 13b. The upconverted emission from the TTA-UC thin films incorporating 20 wt.% plasmonic core–shell–shell structures was more than doubled when irradiated with an LED (Figure 13c), similar to noncoherent indoor light. WO_3_, which has an energy gap of 2.8 eV, could be sensitized with light at 425 nm corresponding to the upconverted emission instead of the photoexcitation wavelength for upconverted emission at 532 nm of light. The primary mechanism for the degradation of acetaldehyde was through the photocatalytic production of OH radicals. Consequently, the incorporation of plasmonic core–shell–shell structures into TTA-UC thin films resulted in enhanced efficiency of acetaldehyde decomposition and increased production of CO_2_ as a decomposition product under 532 nm light irradiation. This demonstrates that plasmonic TTA-UC systems can improve indoor photocatalytic performance (Figure 13d).

**Figure 14:**
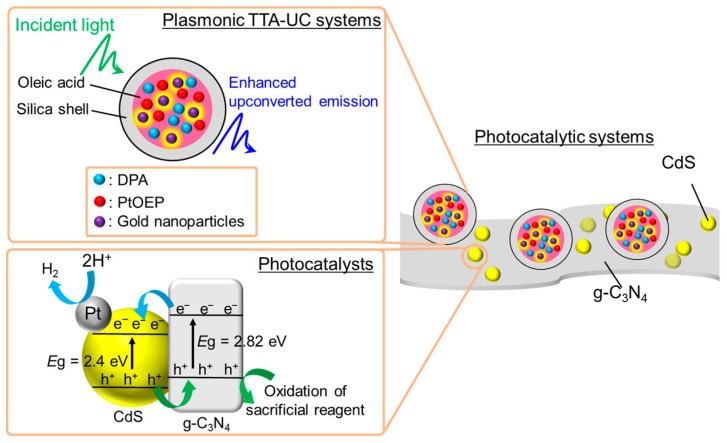
Schematic of plasmonic TTA-UC systems-based photocatalytic systems for hydrogen generation.

Hydrogen has recently garnered attention as a promising and environmentally friendly energy source. Photocatalytic water splitting for hydrogen generation is a particularly attractive approach for addressing global energy needs. Plasmonic TTA-UC systems were utilized to achieve efficient visible-light-driven photocatalytic hydrogen generation [67]. Rigid silica shells encapsulated oleic acid containing PtOEP, DPA, and gold nanoparticles with a diameter of 6 nm in the form of 200 nm spherical capsules, which constituted the plasmonic TTA-UC systems (Figure 14). The upconverted emission was enhanced twice over by the photoexcitation enhancement in PtOEP, which was caused by the LSP resonance of gold nanoparticles. The photocatalytic systems were prepared by conjugating the plasmonic TTA-UC capsules with photocatalytic complexes of CdS nanoparticles-graphitic carbon nitrides (g-C_3_N_4_) to achieve efficient visible-light-driven photocatalytic hydrogen generation. Under irradiation with visible light (420 nm < *λ* < 780 nm), the samples with gold nanoparticles exhibited a 57% higher hydrogen production than those without gold nanoparticles. The plasmon-enhanced upconverted emission markedly improved the efficiency of the photocatalytic reaction processes involved in hydrogen generation. Specifically, the photogenerated holes in the CdS were efficiently captured by g-C_3_N_4_, which facilitated the oxidation of the sacrificial reagent. Furthermore, the photoinduced electron transfer from g-C_3_N_4_ to CdS reduced protons to hydrogen (Figure 14).

**Figure 15:**
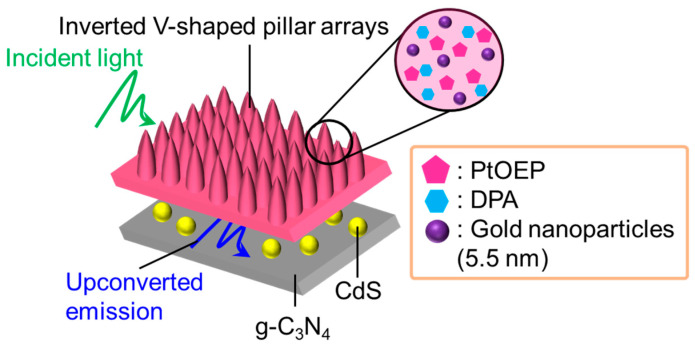
Schematic of photocatalytic systems utilizing photonic crystals–gold nanoparticles coupled systems.

The inverted V-shaped pillar arrays made of polydimethylsiloxane, containing PtOEP, DPA, and 5.5 nm gold nanoparticles, were utilized to further improve the photocatalytic activity by exploiting the coupled effect of photonic crystal structures with plasmonic metal nanoparticles (Figure 15) [68]. The finite-difference time-domain (FDTD) simulations of the pillar arrays exhibited a strong concentration of electromagnetic fields at the top and bottom of the pillars, suggesting their potential to function as photonic crystals for trapping photons. Furthermore, the insertion of gold nanoparticles into the structures further amplified the intensity of the electromagnetic fields, resulting in a synergistic effect that enhances the light-trapping properties of the photonic crystal and the plasmonic properties of the nanoparticles. Moreover, experimental results have demonstrated a significant enhancement in both the upconverted emission and the pseudo-first-order rate constant for photocatalytic degradation of tetracycline by the g-C_3_N_4_-CdS conjugates. Additionally, reduced graphene oxide-CdS nanoparticle conjugates have utilized plasmonic TTA-UC systems for the photocatalytic degradation of tetracycline, as shown in studies [69]. The plasmonic TTA-UC thin solid films, consisting of PtOEP, DPA, and 5.5 nm gold nanoparticles, and polydimethylsiloxane as a matrix host, were sequentially modified with the reduced graphene oxide and CdS nanoparticles to act as a recombination inhibitor and catalyst, respectively. Optimizing the quantity of inserted gold nanoparticles notably enhanced the upconverted emission, resulting in a marked improvement in the pseudo-first-order rate constant of tetracycline. This enhanced photocatalysis was attributed to the efficient sensitization of CdS by the upconverted emission, as described above.

## 4. Outlook

This review introduced the mechanisms behind the enhancement in upconverted emission from plasmonic TTA-UC systems by applying metal-enhanced fluorescence systems. The radiation mechanism of TTA-UC systems differs from that of individual fluorescent molecules. Therefore, other mechanisms for enhancing upconverted emission via LSP resonance must be considered. For instance, recent research has demonstrated that the LSP resonance of silver nanowires enhances the Dexter-type energy transfer between the sensitizer and triplet-excite emitter and between the triplet-excited emitters in TTA-UC systems [70]. Further research is needed to determine the LSP-induced factors that can improve the performance of TTA-UC systems. Although several examples of plasmonic TTA-UC systems were presented here, significant enhancement in upconverted emission is limited due to the inefficient incorporation of TTA-UC elements into plasmonic electromagnetic near-fields. Additionally, the impact of quenching factors, which are not accounted for in metal-enhanced fluorescence, should be taken into consideration as they may impair the upconverted emission enhancement effect. As previously discussed, the energy transfer from the triplet-excited sensitizer to LSP was found to inhibit the TTET process in plasmonic TTA-UC thin solid films. Furthermore, recent studies have reported noteworthy fluorescence from phosphorescent PdOEP by the LSP resonance of triangular silver nanoplates [71]. These findings suggest that the high ISC efficiency of singlet-excited PdOEP can be reduced by competing energy transfer to the LSP resonance. It is crucial to determine the LSP-induced quenching factors characteristic of plasmonic TTA-UC systems for the development of optimized systems that exhibit significant enhancement in upconverted emission. In the near future, the optical interaction between plasmons and upconversion systems may be more comprehensively understood by applying Fermi’s golden rule. Moreover, it is worth noting that plasmonic TTA-UC systems have thus far only utilized Pd(Pt) porphyrins, which have been commonly employed in TTA-UC systems. However, it is important to recognize that the impact of plasmonic electromagnetic near-fields on the molecular photophysical properties likely depends on the original electronic properties of the molecules. Therefore, to advance both fundamental and applied studies, it is necessary to develop plasmonic TTA-UC systems that incorporate the various advanced sensitizers discussed above.

## Data Availability

The data underlying the results presented in this review are not publicly available at this time but may be obtained from the authors upon reasonable request.
